# A Novel Strategy for Emergency Treatment of Coronary Perforations by Placing a Drug-Eluting Stent before Sealing off the Leakage with a Covered Stent to Improve Long-Term Outcomes in Patients with Coronary Artery Perforations

**DOI:** 10.3390/jpm13111542

**Published:** 2023-10-26

**Authors:** Mohamed Ayoub, Noé Corpataux, Péter Tajti, Michael Behnes, Tobias Schupp, Jan Forner, Ibrahim Akin, Dirk Westermann, Volker Rudolph, Kambis Mashayekhi

**Affiliations:** 1 Division of Cardiology and Angiology, Heart Center University of Bochum, 32545 Bad Oeynhausen, Germany; 2 Division of Cardiology, University Hospital Bern, 3010 Bern, Switzerland; noe.corpataux@insel.ch; 3 Division of Interventional Cardiology, Gottsegen Gyorgy National Cardiovascular Center, 1096 Budapest, Hungary; 4 First Department of Medicine, University Medical Centre Mannheim, Faculty of Medicine Manheim, University of Heidelberg, 67059 Heidelberg, Germany; 5 Faculty of Medicine Göttingen, University of Göttingen, 37077 Göttingen, Germany; 6 Division of Cardiology and Angiology II, University Heart Center Freiburg—Bad Krozingen, Faculty of Medicine of the University Freiburg, 79106 Freiburg, Germany; 7 Clinic of Internal Medicine and Cardiology, Heart Center Lahr, 77933 Lahr, Germany

**Keywords:** covered stent, perforation, coronary artery disease

## Abstract

We aimed to investigate the safety, feasibility, and long-term results of drug-eluting stent implantation before covered stents for treating coronary artery perforation (CAP). Between 2015 and 2020, 12,733 patients undergoing percutaneous coronary intervention (PCI) were retrospectively analyzed. The primary endpoint was 1-year target lesion revascularization (TLR), whereas secondary endpoints included the rate of major adverse cardiac and cerebrovascular events (MACCE) and all-cause death at 1 year. A total of 159 patients with CAP were identified during the study period, of whom 47.2% (n = 75) were treated with a covered stent (CS group) because of complex and/or severe CAP and 84 (52.8%) without (non-CS group). In the majority of patients, emergency drug-eluting stent placement before covered stent implantation was feasible (n = 69, 82%). There were no significant differences among patients treated with or without a covered stent in terms of primary or secondary clinical endpoints: a similar rate of TLR (18.67% vs. 21.43%, *p* = 0.6646), MACCE (25.33% vs. 22.62%, *p* = 0.6887), and 1-year mortality (12.00% vs. 11.90%, *p* = 0.9853) were identified comparing cases with covered stent implantation and without. In conclusion, our study implicates that the use of covered stents for sealing coronary perforation might not impact the 1-year clinical outcome if used properly. Moreover, the emergent use of drug-eluting stents before covered stent implantation in CAP is a safe and effective method to avoid target lesion revascularization in patients treated with covered stents.

## 1. Introduction

Coronary artery perforation (CAP) is a rare and feared complication, with a varying incidence ranging from 0.1% to 0.8% of all percutaneous coronary interventions (PCIs) [1,2,3], and up to 4.8–8.9% for complex coronary lesions, such as chronic total occlusions (CTOs) [4,5]. Some other independent factors have been found to increase the risk of CAP, like advanced age, diabetes mellitus, renal dysfunction, and the use of atherectomy devices or cutting balloons due to severe calcification [2,6,7]. After CAP, the 1-year all-cause mortality reaches 18–35%, which is two to three times more than in PCI patients without CAP [2,3,8,9]. The grade of CAP correlates well with the clinical outcome and is usually stratified using the Ellis classification, first described in 1994 [10].

The design of covered stents is unique in that it establishes a barrier between the endothelium of the blood vessel and its lumen. They are considered an effective salvage strategy, and numerous observational studies have shown the superiority of covered stents over prolonged balloon inflation and heparin reversal, predominantly in cases of Ellis type III perforations [3,11,12,13]. Nevertheless, covered stents are known to have a potentially higher rate of in-stent restenosis and stent thrombosis. It has never been investigated whether additional drug-eluting stent placement with abluminal drug delivery before covered stent implantation might be reasonable. Despite the high prevalence of CAP, covered stents are primarily used in emergency indications, and clinical outcome data are most often limited to registries with small sample sizes or missing follow-up data.

Our study presents the outcomes of a large CAP cohort, comparing patients treated with covered stents after drug-eluting stent (DES), implantation (CS group), or without covered stents (non-CS group), including a 1-year follow-up. We sought to investigate whether the use of covered stents in coronary perforation impacts the clinical outcome of the patients and whether the use of DES before covered stents shows a clinical benefit.

## 2. Materials and Methods

### 2.1. Study Population

All consecutive patients undergoing PCI at our center between January 2015 and January 2020 were entered in the hospital database. As part of the quality management program of our institution, baseline demographic, clinical, angiographic, and procedural data, as well as outcome data, were routinely entered in the hospital monitoring database. As part of our routine follow-up, we performed interviews one year and three years after any PCI, and the results were also documented in the database. Written informed consent for PCI was obtained from each patient, and data collection was performed following the Declaration of Helsinki and approved by the institutional review board (University Heart Center Freiburg—Bad Krozingen, Ethical approval number: EK 21-1100). There were no formal exclusion criteria, and all patients who provided informed consent were included. In this single-center retrospective analysis, patients who underwent a CAP during a coronary angiogram or PCI were stratified according to whether a covered stent was used, based on the operator’s discretion. In cases when a covered stent was indicated, a drug-eluting stent was implanted before the covered stent whenever possible to ensure abluminal drug delivery. The majority of patients with chronic coronary syndrome received uploading with clopidogrel. The recommended duration for dual antiplatelet therapy (DAPT) was 12 months for all patients until August 2017, unless there was concomitant oral anticoagulation or high bleeding risk features. From August 2017, the routinely recommended DAPT duration for chronic coronary syndrome patients was six months, according to the current guidelines of the European Society of Cardiology [14].

### 2.2. Definitions

Clinical perforation was defined as any perforation requiring treatment. Subsequent angiography restenosis and stent thrombosis in previously implanted stents was registered.

Procedural success was defined as technical success, indicated by residual stenosis of less than 30%, without any major adverse cardiac and cerebrovascular events during hospital stay.

In-hospital and 1-year major adverse cardiovascular and cerebral event (MACCEs) included any of the following adverse events before hospital discharge and at the 1-year follow-up, respectively: all-cause death, type 4 myocardial infarction (MI) using the Third Universal Definition of Myocardial Infarction [15], stroke, target-vessel revascularization (TVR) or target-lesion revascularization (TLR) with PCI or coronary artery bypass graft (CABG), and tamponade requiring pericardiocentesis. In-stent restenosis was defined as stenosis assessed by angiographic visual estimation by the physician (>50%) or by fractional flow reserve (FFR) ≤ 0.80 in a previously stented segment identified by coronary angiography for any clinical indication. Target lesion revascularisation (TLR) was defined as any attempt to perform another PCI in the same coronary segment as the index procedure. Severe tortuosity was defined by a bend of >90° within the lesion. Severe calcification of the target lesion was defined as cine angiography (i.e., radiopacities noted without cardiac motion before contrast injection, which generally compromised both sides of the arterial lumen) [16].

### 2.3. Statistical Methods

Baseline, procedural, and lesion characteristics are shown as means and standard deviations or as medians with interquartile ranges (IQR), unless otherwise specified. Categorical variables were expressed as percentages and compared using Pearson’s chi-square test or Fisher’s exact test. A *p*-value of *p* < 0.05 was considered statistically significant, and all *p*-values were 2-sided. All statistical analysis was performed with JMP 13.0 (SAS, Cary, NC, USA).

## 3. Results

**Figure 1:**
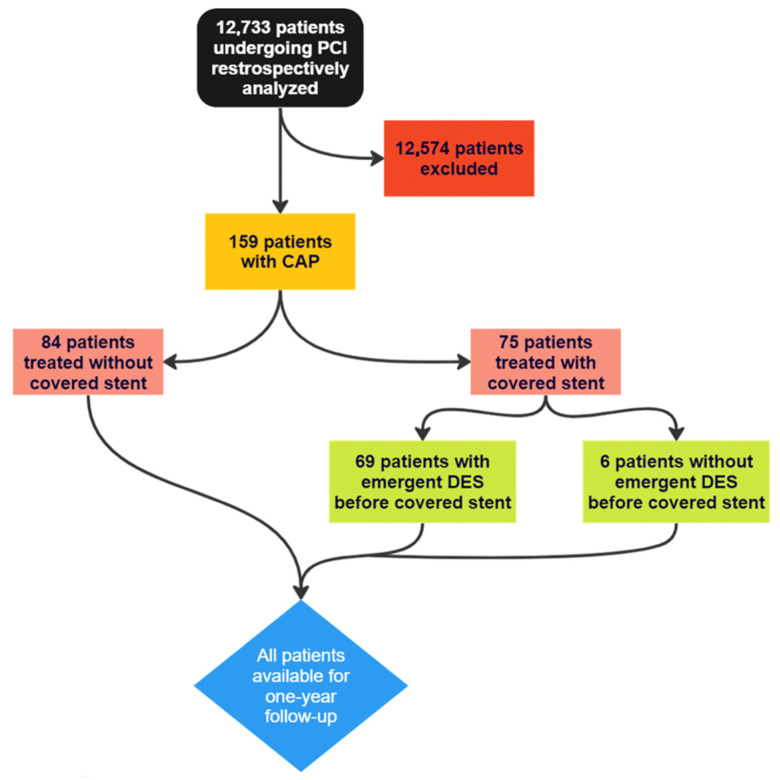
Flowchart of the patients with a therapy strategy.

A total of 159 patients with CAP were identified during the study period, of whom 75 (47.2%) were treated with covered stents and 84 (52.8%) without. An emergent drug-eluting stent placement before covered stent implantation was feasible in most cases requiring covered stents (n = 69). All patients were available for one-year follow-up (Figure 1). The baseline characteristics of the study population are listed in Table 1. The majority of patients were male (70.44%), and the median age was 72.8 ± 9.7 years. Cardiovascular risk factors were common, such as dyslipidaemia (88.36%), diabetes (30.20%), hypertension (90.26%), or a history of coronary artery disease (31.78%). There were no significant differences in baseline clinical features between the study groups, except for the incidence of hypertension, which was significantly higher among patients in the CS group (97.33% vs. 83.54%, *p* = 0.0040).

The lesion characteristics are shown in Table 2, and procedural characteristics are outlined in Table 3. Lesions in the CS group were more complex due to the evidence of more severe calcification (*p* = 0.0084) or a higher incidence of American Heart Association (AHA) Classification B2/C type lesions (*p* = 0.0040). Perforations treated with covered stents were also more severe and stratified by the Ellis classification (Type II–III, 94.02%, *p* = 0.0001). Procedural success was achieved in 83.65% and there were no significant differences between the compared groups (85.33% [CS] and 82.14% [non-CS] *p* = 0.5871). A larger balloon diameter for pre-dilatation was, however, used in the CS group (3.1 ± 1.8 mm vs. 2.5 ± 0.6 mm; *p* = 0.0037), and rotational atherectomy was also more common (26.66% vs. 11.9%; *p* = 0.018) compared to non-CS cases. Procedural times (120 [IQR 85–198] min vs. 86 [IQR 56.3–135] min, *p* = 0.0002) and fluoroscopy times (47.5 [IQR 28.6–98.8] min vs. 27 [IQR 18.5–53.5] min, *p* = 0.0006) were longer in the CS group. Pericardial effusion manifested more often in patients treated with covered stents compared to patients without CS use (38.66% vs. 17.50% *p* = 0.0033). The covered stents used were primarily PTFE and Papyrus, which accounted for 47% of the cases. In the non-CS group, perforations were treated with prolonged balloon inflation or coils (6.92%). The mechanisms for CAP development are listed in Table 4. The most common reasons for CAP in the CS group were severe calcification (44.00%), microcatheter or wire exit (14.67%), or the use of oversized balloon or stent (17.33%). In contrast, the mechanisms of perforation in the non-CS group were slightly different: microcatheter or wire-based perforations were common (30.95%), but in the majority of the cases (51.19%), no clear reason was detected.

### Procedural Outcomes at 1 Year of Follow-Up

**Figure 2:**
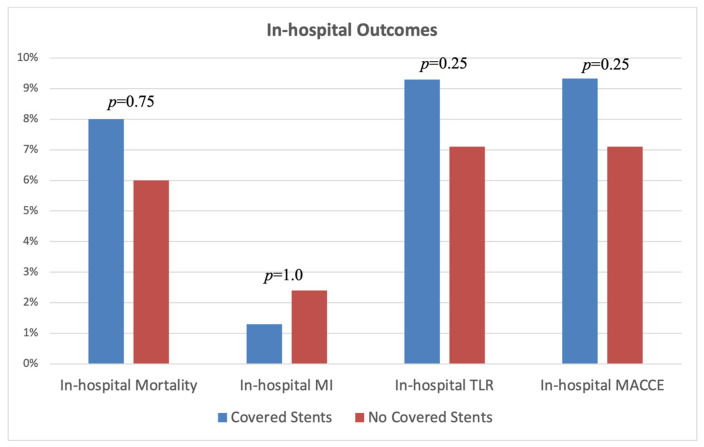
In-hospital clinical outcomes were stratified by whether a covered stent was used or not.

**Figure 3:**
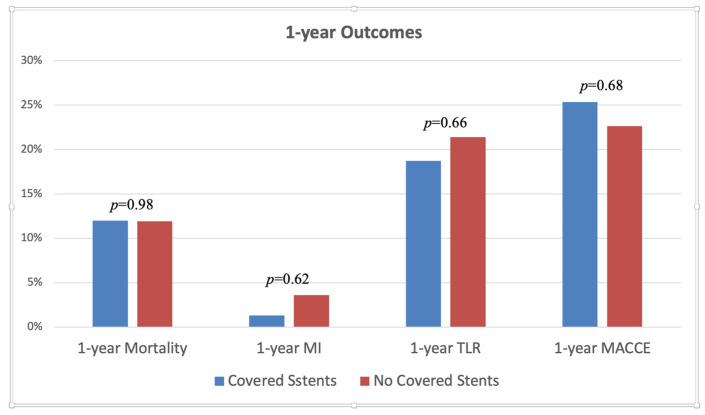
1-year clinical outcomes stratified by whether covered stent was used or not.

The major findings of our study are presented in Table 5, Figure 2 and Figure 3. There were no significant differences between the two study groups regarding the primary or secondary clinical endpoints, representing a similar rate of TLR (18.67% vs. 21.43%, *p* = 0.6646), MACCE (25.33% vs. 22.62%, *p* = 0.6887), and mortality (12.00% vs. 11.90%, *p* = 0.9853) during 1 year of follow-up. Similar findings were observed regarding the in-hospital outcomes (Figure 2). In addition, consistent results were observed in terms of 1-year all-cause death (12.00% vs. 11.90%, *p* = 0.9853) and MI rates (1.33% vs. 3.57%, *p* = 0.6227).

## 4. Discussion

Despite its low incidence, CAP represents a major cause of fatal outcomes in cardiac catheterization and remains a challenge for physicians worldwide. To the best of our knowledge, our study is the largest to date to compare the long-term outcomes of CAP treated with covered stents and without, implementing a novel strategy of emergent drug-eluting stent implantation before covered stent implantation for improving long-term outcomes in patients with coronary perforation. Our main findings can be summarized as follows: (a) covered stents are mostly needed to treat severe CAP (94% Ellis classification Type II-III), (b) severe perforations that required covered stent implantation typically occurred in complex and calcified lesions that needed high pre-dilatation pressure and atherectomy devices, (c) drug-eluting stent implantation before covered stent implantation is safe and does not impact clinical outcomes compared to patients treated without covered stents, (d) to the best of our knowledge, our data has shown the lowest 1-year MACCE rates after covered stent implantation in the current literature [6,15,17,18,19].

It is important to note that in-hospital complications and mortality were not caused by the covered stents but rather by the reasons for their use. However, the high in-hospital mortality of 6.9% reported in our study (8.0% in the covered stent group vs. 5.9% in the non-covered stent group) is much lower than the data published by Harnek et al. in the SCAAR registry [6] (1-month mortality of 12.6% for any CAP), as well as the data from the pooled analysis by Nagaraja et al. (in-hospital mortality of 9.8% by patients treated with covered stents) [20]. This could be explained by the fact that we are reporting data from a high-volume center with skilled operators and because many perforations were linked to CTO procedures or during complex interventions requiring 6 or 7 French guiding catheters, sometimes simultaneously, thus simplifying and accelerating the bailout strategies like the ping-pong technique [21,22]. These techniques make it possible to implant a drug-eluting stent before a covered stent without risking a pericardial tamponade. It is particularly noteworthy that the majority of perforations did not progress to acute tamponade, reflecting the ability of the physicians to treat the perforation immediately and avoid the evolution of such a critical complication leading to hemodynamic instability. 

Covered stents have been used as a treatment option for coronary perforation during percutaneous coronary intervention (PCI). The use of covered stents is effective in sealing the perforation site, preventing further extravasation of blood into the pericardial space. Several studies have investigated the use of covered stents for managing coronary perforation during PCI [17]. Despite the potential benefits of covered stents in the management of coronary perforations, there are also some concerns regarding their use. Covered stents may increase the risk of stent thrombosis and restenosis, in addition to the risk of stent malposition and incomplete coverage of the perforation site. Therefore, the MACCE rate is an important outcome measure in studies of covered stent placement for coronary perforation, as it provides valuable information on the clinical impact of this treatment option. Our study reported a 1-year MACCE rate of 25.33% in patients who underwent covered stent placement for coronary perforation, with a mortality rate of 12%. Other studies have reported higher 1-year MACCE rates. Hernandez et. al. showed a 1-year MACCE rate of 57% in patients who underwent covered stent placement, with a mortality rate of 36% [18]. Lee et al. reported a 1-year mortality rate of 26% after coronary perforation with covered stent implantation [19]. 

It is important to underline the low 1-year MACCE rate after coronary perforation in our study, which did not differ from the group without the need for covered stent implantation. This is mainly driven by the lower TLR rate in the covered stent group. Furthermore, no study has ever shown such low 1-year MACCE rates after covered stent implantation. Our theory behind this is the implantation of drug-eluting stents with abluminal drug delivery before the implantation of a covered stent to reduce the TLR rate. Therefore, DES implantation before covered stent implantation may be reasonable in the majority of cases where covered stents are needed to stop the perforation. 

Our results should be interpreted cautiously, given several limitations. Firstly, they arise from an observational, retrospective single-center study and, as such, may suffer from limited generalizability. Second, there was an absence of a control group with covered stents alone. Third, choosing whether to use a covered stent was left to the operators’ discretion. Fourth, only 1-year follow-up data was available for the study group, limiting the estimation of post-procedural complications during a longer follow-up period. Finally, trained physicians performed the interventions in high-volume CTO centres, restricting extrapolation to less experienced operators and lower-volume programs.

## 5. Conclusions

Using covered stents in CAP is safe and less likely to negatively impact the clinical outcomes in patients treated with covered stents compared to those treated without. Drug-eluting stent (DES) implantation before covered stents, if possible, in cases of clinically significant coronary perforation, is feasible and demonstrates relatively low in-hospital and 1-year MACCE-rates post-PCI. However, our findings need to be strengthened in future studies, which should include longer follow-ups and a larger number of cases.

## Figures and Tables

**Table 1:** Baseline characteristics in patients with coronary artery perforation (CAP).

	Overall (n = 159)	Covered Stents (n = 75)	No Covered Stents (n = 84)	*p* Value
Age (years)	72.8 ± 9.7	74.3 ± 8.7	71.4 ± 10.4	0.0581
Men	70.44% (112)	66.67% (50)	73.81% (62)	0.3245
BMI (kg/m^2^)	26.4 ± 3.6	26.3 ± 3.8	26.5 ± 3.5	0.7756
eGFR	64.7 ± 20.3	62.7 ± 20.5	66.5 ± 20.1	0.2623
Class angina (CCS > II)	60.58% (83)	65.67% (44)	55.71% (39)	0.2332
CAD presentation				0.7197
ACS	20.13% (32)	21.33%	19.05%	
No ACS	79.87% (127)	78.66%	80.95%	
Diabetes mellitus	30.20% (45)	22.54% (16)	37.18% (29)	0.0518
Dyslipidemia	88.36% (129)	83.10% (59)	93.33% (70)	0.0540
Hypertension	90.26% (139)	97.33% (73)	83.54% (66)	0.0039
Smoking (current)	12% (18)	12.33% (9)	11.69% (9)	0.9040
LVEF (%)				0.9555
>51%	56.86% (87)	56.00% (42)	57.69% (45)	
41–51%	28.76% (44)	30.66% (23)	26.92% (21)	
30–40%	8.50% (13)	8.00% (6)	8.97% (7)	
0–29%	5.88% (9)	5.33% (4)	6.41% (5)	
History of familiar CAD	31.78% (41)	30.65% (19)	32.84% (22)	0.7895
Prior MI	30.56% (44)	37.68% (26)	24.00% (18)	0.0750
Prior CABG	20.00% (29)	26.09% (18)	14.47% (11)	0.0808
LDL max.	100.1 ± 39.0	97.5 ± 35.6	102.5 ± 41.8	0.419

ACS = acute coronary syndrome; BMI = body mass index; CABG = coronary artery bypass graft surgery; CAD = coronary artery disease; CCS = Canadian Cardiovascular Society; eGFR = estimated glomerular filtration rate in mL/min/1.73 m^2^; LDL = low-density lipoprotein in mg/dL LVEF = left ventricular ejection fraction; MI = myocardial infarction.

**Table 2:** Lesion characteristics in patients with coronary artery perforation (CAP).

	Overall (n = 159)	Covered Stents (n = 75)	No Covered Stents (n = 84)	*p* Value
Target coronary vessel				<0.0001
Left main	5.66% (9)	4.00% (3)	7.14% (6)	
Right	47.17% (75)	56.00% (42)	39.29% (33)	
Left circumflex	11.32% (18)	8.00% (6)	14.29% (12)	
Left anterior descending	35.22% (56)	30.67% (23)	39.29% (33)	
SVG	0.63% (1)	1.33% (1)	0.00% (0)	
Number of treated vessels	1.4 ± 0.7	1.4 ± 0.65	1.5 ± 0.7	0.3076
Lesion length (mm)				0.3149
<10 mm	10.60% (16)	8.45% (6)	12.50% (10)	
10–20 mm	32.45% (49)	28.17% (20)	36.25% (29)	
>20 mm	56.95% (86)	63.38% (45)	51.25% (41)	
Pre-PCI stenosis, %	87.1 ± 17.9	87.1 ± 18.6	87.1 ± 17.2	0.9936
AHA/ACC classification				0.0040 *
A/B1	12.58% (20)	4.00% (3)	20.24% (17)	
B2	20.13% (32)	22.66% (17)	17.86% (15)	
C	67.30% (107)	73.33% (55)	61.90% (52)	
Ostial lesion	3.8% (6)	4.00% (3)	3.57% (3)	1.0000
Calcification				0.0084
None	5.06% (8)	2.67% (2)	7.23% (6)	
Mild	21.52% (34)	12.00% (9)	30.12% (25)	
Moderate	22.78% (36)	22.67% (17)	22.89% (17)	
Severe	50.63% (80)	62.67% (47)	39.76% (33)	
Excentric calcification	66.67% (98)	64.29% (45)	68.83% (53)	0.5593
Severe tortuosity	17.83% (28)	17.33% (13)	18.29% (15)	0.8754
Relevant side branch	29.03% (45)	27.03% (20)	30.86% (25)	0.5991
Intra-lesion Angulation				0.6127
none	17.72% (28)	21.33% (16)	14.46% (12)	
<45%	40.51% (64)	36.00% (27)	44.58% (37)	
45–90%	32.91% (52)	33.33% (25)	32.53% (27)	
>90%	8.86% (14)	9.33% (7)	8.43% (7)	

AHA/ACC = American College of Cardiology/American Heart Association; PCI = percutaneous coronary intervention; SVG = saphenous vein graft, * = statistically significant with *p* < 0.05.

**Table 3:** Technical and procedural characteristics in patients with coronary artery perforation (CAP).

	Total (n = 159)	Covered Stents (n = 75)	No Covered Stents (n = 84)	*p* Value
Technical success	89.94% (143)	93.33% (70)	86.90% (73)	0.1786
Procedural success	83.65% (133)	85.33% (64)	82.14% (69)	0.5871
Balloon diameter predilatation (mm)	2.8 ± 1.3	3.1 ± 1.8	2.5 ± 0.6	0.0037 *
Maximum predilation pressure (atm)	17.5 ± 6.1	18.7 ± 7.6	16.5 ± 4.9	0.1037
Number of stents implanted	1.6 ± 1.1	1.7 ± 1.2	1.5 ± 1.2	0.2546
Diameter of implanted stent (mm)	3.2 ± 0.6	3.31 ± 0.6	3.1 ± 0.5	0.2376
DES implanted before covered stent	69.0%	92%	-	1.0000
Diameter of covered stents	3.4 ± 0.7	3.4 ± 0.7	-	1.0000
Length of covered stents	23.3 ± 14.2	23.34 ± 14.2	-	1.0000
Balloon diameter postdilatation (mm)	3.9 ± 0.8	4.3 ± 0.5	3.4 ± 0.5	0.3966
Postdilatation pressure (atm)	19.5 ± 5.1	20.1 ± 6.5	19.0 ± 3.3	0.7127
Rotational atherectomy	18.87% (30)	26.66% (20)	11.90% (10)	0.0176
TIMI flow post PCI				0.1287
TIMI 0/TIMI I	11.04% (17)	6.94% (5)	14.63% (12)	
TIMI II/TIMI III	88.96% (137)	93.06% (67)	85.37% (70)	
Access site				0.4636
Radial access	38.89% (56)	43.28% (29)	35.06% (27)	
Femoral access	61.11% (88)	56.72% (38)	64.94% (50)	
Guiding catheter size, Fr				0.1890
6	60.53% (92)	48.57% (34)	70.73% (58)	
7	34.21% (52)	44.29% (31)	25.61% (21)	
8	5.26% (8)	7.14% (5)	3.66% (3)	
CTO target vessel	44.30% (70)	52.0% (39)	37.35% (31)	0.0641
CTO technique used				0.5319
antegrade	57.14% (40)	53.85% (21)	61.29% (19)	
retrograde	42.86% (30)	46.15% (18)	38.71% (12)	
Procedural time (min)	101 (71, 163)	120 (85, 198)	86 (56.3, 135)	0.0002
Fluoroscopy time (min)	35 (21, 71)	47.5 (28.6, 98.8)	27.0 (18.5, 53.5)	0.0006
Fluoroscopic Dose Area Product (cGy*cm^2^)	10,537 (5720, 17,898)	13,968 (7666, 22,075)	8210 (4779, 15,553)	0.0034
Contrast volume used (mL)	300 (190, 400)	325 (200, 407.5)	260 (177.5, 380)	0.0803
Ellis classification				0.0001
I	7.75% (16)	4.48% (3)	10.67% (8)	
II	34.51% (49)	38.81% (26)	30.67% (23)	
III	33.10% (47)	55.22% (37)	13.33% (10)	
Unknown	24.65% (35)	1.49% (1)	45.33% (34)	

CTO = chronic total occlusion; DES = drug-eluting stent; PCI = percutaneous coronary intervention; TIMI = thrombolysis in myocardial infarction, * = statistically significant with *p* < 0.05.

**Table 4:** Reason for coronary artery perforation.

	Overall (n = 159)	Covered Stents (n = 75)	No Covered Stents (n = 84)	*p* Value
Not clear	30.82% (49)	8.00% (6)	51.19% (43)	<0.0001 *
Wire or microcatheter exit	23.27% (37)	14.67% (11)	30.95% (43)	
Severe calcification	23.90% (38)	44.00% (33)	5.95% (26)	
Subintimal stenting	1.9% (3)	4.00% (3)	0.00% (0)	
Oversized balloon or stent	10.7% (17)	17.33% (13)	4.76% (4)	
Collateral perforation	3.14% (5)	1.33% (1)	4.76% (4)	
Tortuosity with calcification	6.3% (10)	10.67% (8)	2.38% (2)	

* = statistically significant with *p* < 0.05.

**Table 5:** 1-year clinical outcomes stratified by whether covered stent was used or not.

	Overall (n = 159)	Covered Stents (n = 75)	No Covered Stents (n = 84)	*p* Value
In-hospital MACCE	8.18% (13)	9.33% (7)	7.14% (6)	0.6148
1-year Mortality	11.95% (19)	12.00% (9)	11.90% (10)	0.9853
1-year MI	2.52% (4)	1.33% (1)	3.57% (3)	0.6227
1-year TLR	20.13% (32)	18.67% (14)	21.43% (18)	0.6646
1-year stroke	1.26% (2)	2.67% (2)	0.00% (0)	0.2209
1-year MACCE	23.90% (38)	25.33% (19)	22.62% (19)	0.6887
Pericardiocentesis	22.58% (35)	29.33% (22)	16.25% (13)	0.0516
Myocardial hematoma	1.96% (3)	2.74% (2)	1.25% (1)	0.6059

MACCE = major adverse cardiovascular and cerebral events; MI = myocardial infarction; TLR = target lesion revascularization.

## Data Availability

The datasets used and/or analyzed during the current study are available from the corresponding author upon reasonable request.

## References

[1] Fasseas P., Orford J.L., Panetta C.J., Bell M.R., E Denktas A., Lennon R.J., Holmes D.R., Berger P.B. (2004). Incidence, correlates, management, and clinical outcome of coronary perforation: Analysis of 16,298 procedures. Am. Heart J..

[2] Kinnaird T., Kwok C.S., Kontopantelis E., Ossei-Gerning N., Ludman P., deBelder M., Anderson R., Mamas M.A. (2016). Incidence, Determinants, and Outcomes of Coronary Perforation During Percutaneous Coronary Intervention in the United Kingdom Between 2006 and 2013: An Analysis of 527 121 Cases From the British Cardiovascular Intervention Society Database. Circ. Cardiovasc. Interv..

[3] Guttmann O.P., Jones D.A., Gulati A., Kotecha T., Fayed H., Patel D., Crake T., Ozkor M., Wragg A., Smith E.J. (2017). Prevalence and outcomes of coronary artery perforation during percutaneous coronary intervention. EuroIntervention.

[4] Hirai T., Nicholson W.J., Sapontis J., Salisbury A.C., Marso S.P., Lombardi W., Karmpaliotis D., Moses J., Pershad A., Wyman R.M. (2019). A Detailed Analysis of Perforations During Chronic Total Occlusion Angioplasty. JACC Cardiovasc. Interv..

[5] Tajti P., Burke M.N., Karmpaliotis D., Alaswad K., Werner G.S., Azzalini L., Carlino M., Patel M., Mashayekhi K., Egred M. (2018). Update in the Percutaneous Management of Coronary Chronic Total Occlusions. JACC Cardiovasc. Interv..

[6] Harnek J., James S., Lagerqvist B. (2019). Coronary Artery Perforation and Tamponade-Incidence, Risk Factors, Predictors and Outcomes From 12 Years’ Data of the SCAAR Registry. Circ. J..

[7] Shaukat A., Tajti P., Sandoval Y., Stanberry L., Garberich R., Nicholas Burke M., Gossl M., Henry T., Mooney M., Sorajja P. (2019). Incidence, predictors, management and outcomes of coronary perforations. Catheter. Cardiovasc. Interv..

[8] Shimony A., Joseph L., Mottillo S., Eisenberg M.J. (2011). Coronary artery perforation during percutaneous coronary intervention: A systematic review and meta-analysis. Can. J. Cardiol..

[9] Lemmert M.E., van Bommel R.J., Diletti R., Wilschut J.M., de Jaegere P.P., Zijlstra F., Daemen J., Van Mieghem N.M. (2017). Clinical Characteristics and Management of Coronary Artery Perforations: A Single-Center 11-Year Experience and Practical Overview. J. Am. Heart Assoc..

[10] Ellis S.G., Ajluni S., Arnold A.Z., Popma J.J., Bittl J.A., Eigler N.L., Cowley M.J., Raymond R.E., Safian R.D., Whitlow P.L. (1994). Increased coronary perforation in the new device era. Incidence, classification, management, and outcome. Circulation.

[11] Secco G.G., Serdoz R., Kilic I.D., Caiazzo G., Mattesini A., Parisi R., De Luca G., Pistis G., Marino P.N., Di Mario C. (2016). Indications and immediate and long-term results of a novel pericardium covered stent graft: Consecutive 5 year single center experience. Catheter. Cardiovasc. Interv..

[12] Al-Lamee R., Ielasi A., Latib A., Godino C., Ferraro M., Mussardo M., Arioli F., Carlino M., Montorfano M., Chieffo A. (2011). Incidence, predictors, management, immediate and long-term outcomes following grade III coronary perforation. JACC Cardiovasc. Interv..

[13] Ly H., Awaida J.P., Lesperance J., Bilodeau L. (2005). Angiographic and clinical outcomes of polytetrafluoroethylene-covered stent use in significant coronary perforations. Am. J. Cardiol..

[14] Valgimigli M., Bueno H., Byrne R.A., Collet J.P., Costa F., Jeppsson A., Juni P., Kastrati A., Kolh P., Mauri L. (2018). 2017 ESC focused update on dual antiplatelet therapy in coronary artery disease developed in collaboration with EACTS: The Task Force for dual antiplatelet therapy in coronary artery disease of the European Society of Cardiology (ESC) and of the European Association for Cardio-Thoracic Surgery (EACTS). Eur. Heart J..

[15] Kawamoto H., Tanaka K., Ruparelia N., Takagi K., Yabushita H., Watanabe Y., Mitomo S., Matsumoto T., Naganuma T., Fujino Y. (2015). Short-Term and Long-Term Outcomes After Polytetrafluoroethylene-Covered Stent Implantation for the Treatment of Coronary Perforation. Am. J. Cardiol..

[16] Mintz G.S., Popma J.J., Pichard A.D., Kent K.M., Satler L.F., Chuang Y.C., Ditrano C.J., Leon M.B. (1995). Patterns of calcification in coronary artery disease. A statistical analysis of intravascular ultrasound and coronary angiography in 1155 lesions. Circulation.

[17] Pavani M., Cerrato E., Latib A., Ryan N., Calcagno S., Rolfo C., Ugo F., Ielasi A., Escaned J., Tespili M. (2018). Acute and long-term outcomes after polytetrafluoroethylene or pericardium covered stenting for grade 3 coronary artery perforations: Insights from G3-CAP registry. Catheter. Cardiovasc. Interv..

[18] Hernandez-Enriquez M., Lairez O., Campelo-Parada F., Lhermusier T., Bouisset F., Roncalli J., Elbaz M., Carrie D., Boudou N. (2018). Outcomes after use of covered stents to treat coronary artery perforations. Comparison of old and new-generation covered stents. J. Interv. Cardiol..

[19] Lee W.C., Hsueh S.K., Fang C.Y., Wu C.J., Hang C.L., Fang H.Y. (2016). Clinical Outcomes Following Covered Stent for the Treatment of Coronary Artery Perforation. J. Interv. Cardiol..

[20] Nagaraja V., Schwarz K., Moss S., Kwok C.S., Gunning M. (2020). Outcomes of patients who undergo percutaneous coronary intervention with covered stents for coronary perforation: A systematic review and pooled analysis of data. Catheter. Cardiovasc. Interv..

[21] Silver K.H., Bauman W.B., Berkovitz K.E. (2003). Dual-catheter covered stenting: A novel approach to the treatment of large coronary artery perforations. J. Invasive Cardiol..

[22] Gupta H., Kaur N., Sharma Y., Lim S.T. (2021). Modified double guiding catheter ‘Ping Pong’ technique to treat large coronary perforation: A case report. Eur. Heart J. Case Rep..

